# Artificial Intelligence System for Automatic Mammary Region Extraction Using Semi-subjective Corrected Region for Breast Composition Evaluation

**DOI:** 10.7759/cureus.80545

**Published:** 2025-03-13

**Authors:** Sachi Ishizuka, Chiharu Kai, Tsunehiro Ohtsuka, Hitoshi Futamura, Naoki Kodama, Satoshi Kasai

**Affiliations:** 1 Graduate School of Health and Welfare, Niigata University of Health and Welfare, Niigata, JPN; 2 Department of Radiological Technology, Faculty of Medical Technology, Niigata University of Health and Welfare, Niigata, JPN; 3 Department of Breast Surgery, Ohtsuka Breastcare Clinic, Tokyo, JPN; 4 Department of Breast Imaging, Konica Minolta, Inc., Tokyo, JPN

**Keywords:** artificial intelligence (ai), breast composition, mammary gland regions, mammogram, semi-subjective corrected regions

## Abstract

Introduction

Recently, breast composition has been used as a clinical indicator for breast cancer. Although systems have been developed for objectively extracting mammary gland regions, relying on subjective judgment to identify correct mammary gland regions for breast composition can lead to significant inter-judgment variation. In this study, we automatically extracted mammary gland regions using semi-subjective corrected regions that extract only mammary gland regions while simultaneously determining quantitative regions and examining whether extracted results could be used clinically.

Methods

We used 670 mammograms (Pe-ru-ru, Canon Medical Systems Corporation, Tochigi, Japan). A breast physician with 30 years of experience reading mammograms subjectively evaluated mammary gland regions based on the quantitatively determined regions. We defined these images as semi-subjective corrected region images. Further, we used U-Net for segmentation and the dice coefficient as the evaluation index for the region extraction accuracy. The parameters of U-Net (number of downsampling layers, learning rate, and batch size) and the orientation of input images were changed to improve accuracy. In addition, we calculated the dice coefficient based on the breast composition type to evaluate the clinical usefulness of this study.

Results

The average dice coefficient with the highest accuracy was 0.882; the average dice coefficients were 0.992, 0.832, 0.904, and 0.943 for fatty, scattered, heterogeneous dense, and extremely dense regions, respectively.

Conclusion

The mammary gland region was automatically extracted using semi-subjective corrected region images. The average dice coefficients for the whole breast and for each breast composition were high, suggesting that this method is clinically useful.

## Introduction

Breast cancer is the most frequently diagnosed women’s cancer worldwide, and its incidence is gradually increasing [1,2]. Breast cancer screening is performed via mammography, which is the only modality proven to reduce breast cancer mortality in randomized controlled trials [3,4]. Mammograms illustrate the differences in tissue X-ray absorption as density variations; however, the difference in X-ray absorption between mammary and cancer tissues is similar; therefore, both tissues show similar brightness on the mammogram. Therefore, the sensitivity of lesion detection depends on the amount of mammary tissues [5,6]. Breast composition determines the sensitivity of lesion detection during breast cancer screening as indicated in the guidelines established in February 2020 by the Japan Central Organization on Quality Assurance of Breast Cancer Screening. This classification is referenced on a global standard guideline, the Breast Imaging Reporting and Data System (BI-RADS) Atlas developed by the American College of Radiology (ACR) [7]. Breast composition is evaluated using the mammary gland content ratio, which represents the ratio of the region where the mammary gland tissue is believed to have originally existed (excluding the fat-only portion of the obvious postmammary gap, subcutaneous fat, and pectoralis major muscle) as the denominator and sum of regions in the denominator spread equal or greater in density compared to the pectoralis major muscle as the numerator (hereafter, the area of the numerator is referred to as the “mammary gland region”). The breast composition is classified into four categories based on the calculated mammary gland content separated by a threshold value. Regions with mammary gland content of less than 10%, between 10% and 50%, between 50% and 80%, and ≥80% are classified as “fatty,” “scattered,” “heterogeneous dense,” and “extremely dense” regions, respectively [8,9]. Further, “fatty” and “scattered” regions with mammary gland content of <50% are defined as “fatty breast.” “Heterogeneous dense” and “extremely dense” regions with a mammary gland content of >50% are defined as “dense breast.” Breast composition is used as an indicator for assessing the risk of breast cancer and the reference of additional ultrasound in breast cancer screening because “dense breast” has been reported to have lower sensitivity in detecting lesions on mammography and an increased risk of developing breast cancer [1,4,10-13].

In March 2023, the US Food and Drug Administration mandated that facilities offering mammography-based breast cancer screening inform patients of their breast composition [14,15]. It has actually been in effect in the United States since September 2024 [16,17]. Due to the increasing global demand for breast composition, determining the mammary gland content ratio has become indispensable, which in turn requires the extraction of the mammary gland region. However, mammary gland regions are extracted by determining the subjective judgment of the reader, and the region to be extracted varies depending on the clinical experience, skill, and reading environment of the reader. Breast composition judgments exhibit variations among readers, and low inter-reader agreement has posed a significant challenge [9,13,18,19].

Recently, artificial intelligence (AI) has been used to classify breast composition automatically [12,20,21]. Moreover, techniques have been developed for segmenting the mammary gland regions [19,22]. AI-extracted mammary gland regions are presented to the reader for visual convincing. Several studies focused on the consistency between subjective judgment by radiologists and AI [23-25]. In those papers, the correct mammary gland regions are determined by the subjective judgment of one or more readers. Subjectively determining the correct mammary gland regions suggests that the correct mammary gland regions can be determined based on the intuition of the reader by distinguishing between the mammary gland regions and blood vessels or abnormal findings such as calcifications. The range of correct mammary gland regions can differ among readers, thereby resulting in a large degree of variation. Also, evaluating a large number of cases is difficult because determining the correct range of mammary gland regions is time-consuming. The advantage of objectively determining the correct mammary gland regions is that these regions are quantitative and less variable. However, the disadvantage, as typified by threshold processing, is that the pectoralis major muscle, blood vessels, and abnormal findings, which have the same brightness as the mammary tissue, can be judged as mammary tissue and be incorrectly included in the mammary gland regions. Quantitatively determining mammary gland regions and extracting only the regions necessary for breast composition determination simultaneously are necessary for solving the abovementioned problems. Further, automatic mammary region extraction based on Japanese guidelines established in 2020 (hereafter referred to as “new guidelines”) is yet to be evaluated.

Therefore, we aimed to establish quantitative and accurate ground truth (correct mammary gland regions) by introducing the subjective judgment of the reader to images that underwent objective threshold processing. The correct mammary gland region was defined as a semi-subjective corrected region. Determining quantitative regions and simultaneously extracting only mammary tissues by creating semi-subjective corrected regions can help reduce variation. We automatically extracted mammary gland regions based on new guidelines and examined whether extracted results could be used clinically using this semi-subjective corrected region. This study should improve the accuracy and efficiency of the work involved in creating the correct answer region.

## Materials and methods

This study was conducted as follows: data selection, the creation of semi-subjective correct regions, segmentation by U-Net, and the evaluation of extraction accuracy using dice coefficients.

Data selection

**Figure 1:**
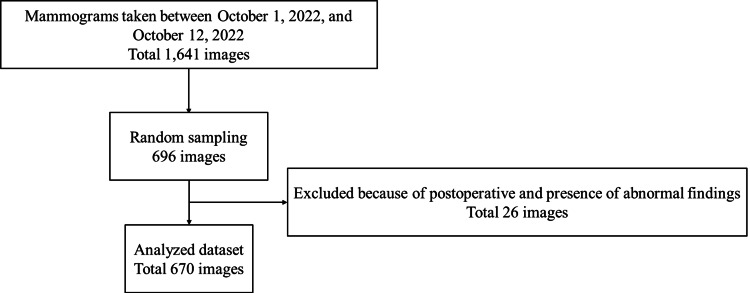
Flow of data selection.

The Institutional Review Board of the Niigata University of Health and Welfare approved this study (approval number: 19109-230717). In this study, we used mammograms obtained from the mediolateral oblique (MLO) orientation recommended by the guidelines. We randomly selected 696 images (354 patients) from 1,641 images (835 patients) obtained using mammograms (Pe-ru-ru, Canon Medical Systems Corporation, Tochigi, Japan) at the Ohtsuka Breastcare Clinic between October 1 and 12, 2022. The mammograms used in this study were collected by Konica Minolta, Inc. and shared as anonymized processed information without personal information. Based on the judgment of the breast physician, 26 of the 696 images were excluded. The exclusion criteria were postoperative or abnormal findings (mass or calcification). A flowchart of the data selection process is shown in Figure 1.

Creation of semi-subjective corrected region images

**Figure 2:**
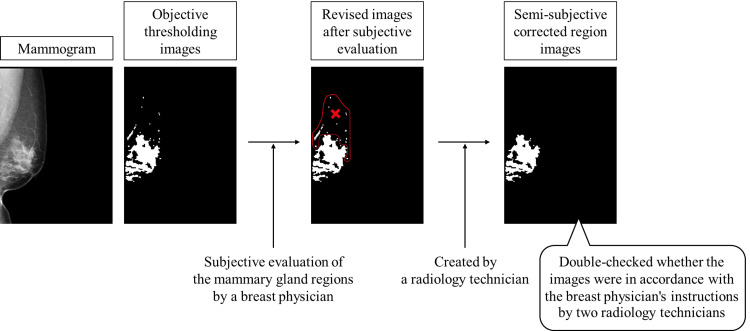
Flow of creating semi-subjective corrected region images.

As objective thresholding images, mammary gland regions on the mammogram were extracted using the threshold method. The threshold method involves the following steps: we created a histogram of the pixel values in the calculated region to determine the mammary gland regions in the mammogram. The threshold was determined by the maximum entropy method when the created histogram had a single peak, and the threshold was determined by the discriminant analysis method when the histogram had two or more peaks [26]. This technology was developed by Konica Minolta, Inc. Next, a breast physician with 30 years of experience reading mammograms evaluated the mammary gland regions (subjective evaluation) based on the objective thresholding images. By performing the subjective evaluation, regions other than blood vessels and mammary gland regions that had been extracted in the objective thresholding images were deleted, and only the mammary gland regions used for breast composition determination were extracted. Based on the 670 images evaluated by the breast physician, a radiology technician created semi-subjective corrected region images. Finally, two radiology technicians double-checked whether the semi-subjective corrected images were in accordance with the breast physician’s instructions. Figure 2 shows the flow of creating semi-subjective corrected region images. The breast physician judged 14 of the 670 images as having no mammary gland regions (hereafter referred to as “unmasked images”).

The mammograms were classified randomly into 530, 70, and 70 images for training, validation, and testing, respectively. Table 1 lists a breakdown of the datasets and breast compositions. The breast composition was determined at the data collection facility. Unmasked images included 10 images in the “scattered” for train, two images in the “scattered” for validation, and two images in the “fatty” for test.

**Table 1:** Breakdown of datasets.

	Train	Validation	Test
Images (pairs)	530	70	70
Breast composition			
Fatty	5	0	2
Scattered	185	29	26
Heterogeneous dense	315	41	38
Extremely dense	25	0	4

Konica Minolta, Inc. was involved in the collection of mammograms and the creation of objective thresholding images; however, it was not involved in the study design, analysis, model development, or manuscript preparation.

Segmentation method

**Figure 3:**
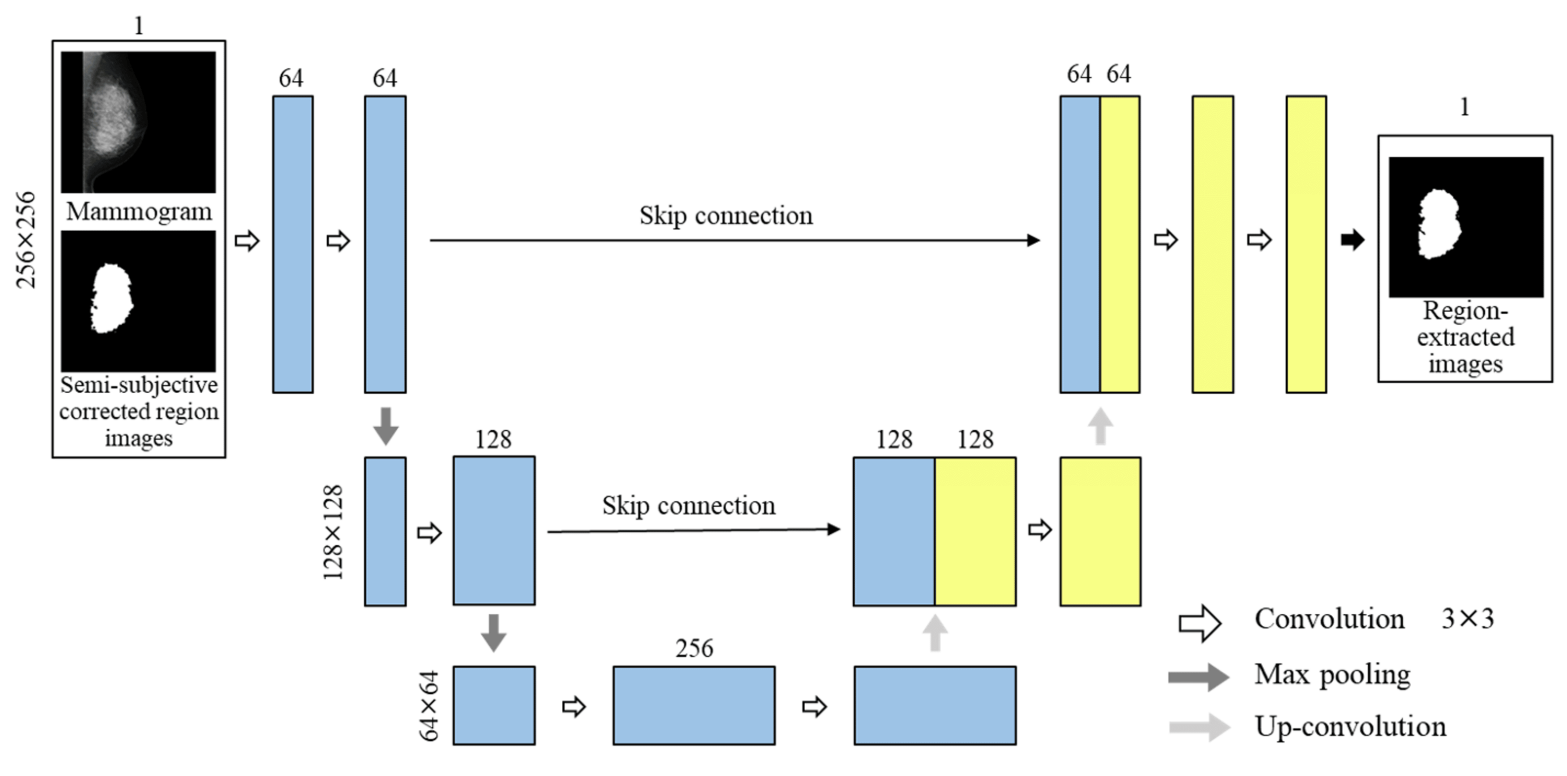
Structure of U-Net.

In this study, U-Net is used as the segmentation technique [27]. Mammograms were input to U-Net, and the mammary gland regions were trained based on semi-subjective corrected region images. Output images were defined as region-extracted images. U-Net was built using a Neural Network Console, which is an integrated development environment for deep learning developed by Sony Corporation (Tokyo, Japan) [28]. When inputting the mammograms into the U-Net, the density resolution was changed from 14 to eight bits, and the image size was resized from 2,016×2,816 to 256×256 using the nearest neighbor interpolation method. Padding was applied to both edges of the images. Further, semi-subjective corrected region images were also resized and padded, similar to the mammograms. The structure of the U-Net is shown in Figure 3. The number of downsampling layers (three, four, five, six, and seven), learning rate (0.001 and 0.0001), and batch size (16 and 32) were considered as parameters of the U-Net. The number of convolution kernels was 64 for the first downsampling layer, and each additional layer was configured to increase twice from those of the previous downsampling layer. Four datasets with different input image orientations were prepared for U-Net with the highest accuracy parameters, and the orientation of the input images was examined. Dataset 1 shows mammograms and semi-subjective corrected region images aligned to the left, whereas Dataset 2 shows the mammogram and semi-subjective corrected region images with the left breast facing left on the image and the right breast facing right. Dataset 3 is the case of the image augmentation of mammograms with random proportions from Dataset 1. Dataset 4 is the case of the image augmentation of mammograms with random proportions from Dataset 2. We used a computer with an 11th Gen Intel(R) Core(TM) i9-11900K @ 3.50 GHz CPU and an NVIDIA GeForce RTX 3 GPU with 64 GB of main memory.

Evaluation method

The dice coefficient, which indicates the degree of similarity, was used as an index to compare the accuracy of region extraction between the semi-subjective corrected region image and region-extracted images [29]. This is an evaluation index used in many segmentation studies [23-25]. The dice coefficient was calculated using the following formula:

$\text{Dice} = \frac{2 |X \cap Y|}{|X| + |Y|}$

**Figure 4:**
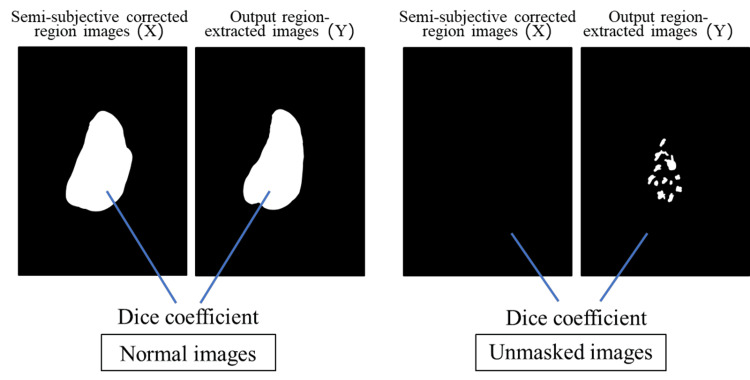
Images of X and Y used to calculate dice coefficients.

The dice coefficient ranged from zero to one. The closer the value is to one, the higher the similarity between X and Y (in this study, the agreement of region extraction), thereby indicating a higher learning effect. In this study, X and Y indicate the semi-subjective corrected region image and region extraction image output by U-Net, respectively. Images of X and Y used in this study to calculate the dice coefficients are presented in Figure 4. The dice coefficient of the white regions in the output region-extracted images (Y) was calculated for the white region in the semi-subjective corrected region images (X). For the unmasked images, the dice coefficient of the black region in the output region-extracted images (Y) was calculated for the black region in the semi-subjective corrected region images (X). We evaluated each parameter combination and dataset three times, and the average of the dice coefficients was calculated and compared. In addition, we calculated the dice coefficients for each breast composition.

## Results

Parameter adjustment

Table 2 lists the average dice coefficient when adjusting U-Net parameters by changing the number of downsampling layers (three, four, five, six, and seven), learning rate (0.001 and 0.0001), and batch size (16 and 32) and using all combinations. Table 3 lists the results of studying the orientation of the input image using U-Net for parameters with the highest average dice coefficients.

**Table 2:** Comparison of the dice coefficient by all parameters.

Number of downsampling layers	Learning rate	Batch size	
		16	32
3	0.001	0.738	0.724
	0.0001	0.769	0.820
4	0.001	0.812	0.829
	0.0001	0.835	0.846
5	0.001	0.865	0.870
	0.0001	0.867	0.864
6	0.001	0.865	0.867
	0.0001	0.873	0.872
7	0.001	0.863	0.872
	0.0001	0.872	0.866

**Table 3:** Comparison of the dice coefficient for dataset orientations.

Dataset	Average dice coefficient
1	0.877
2	0.877
3	0.882
4	0.621

Dice coefficient for each breast composition

**Figure 5:**
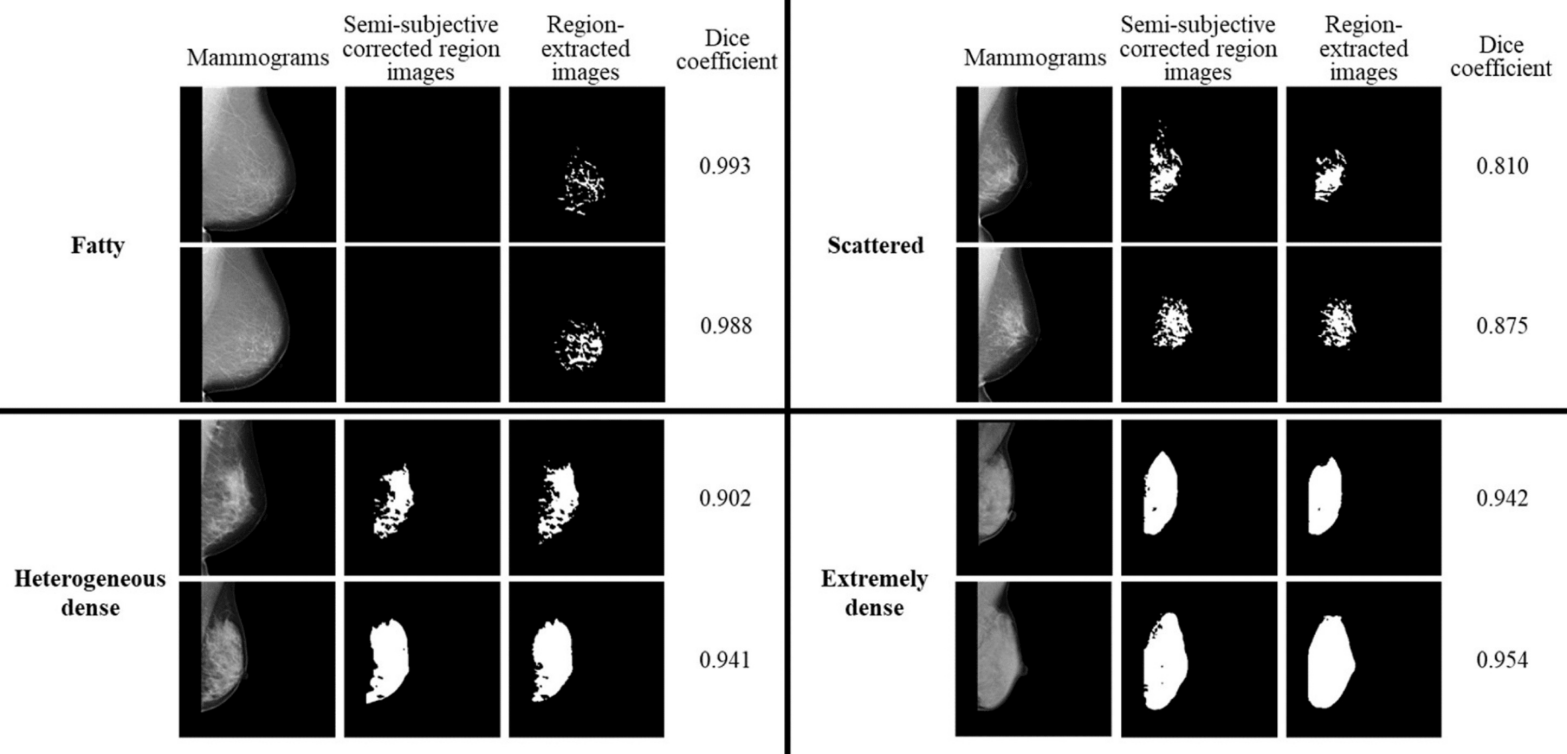
Comparison of the results for each breast composition.

The parameters of U-Net with the highest average dice coefficient included six layers of downsampling, a learning rate of 0.0001, and a batch size of 16. Table 4 summarizes the dice coefficients for each breast composition in the results using Dataset 3 as the input images for U-Net. We confirmed the characteristics of the extracted region for each breast composition. Figure 5 shows the mammograms, semi-subjective corrected region images, and region-extracted images for each breast composition.

**Table 4:** Dice coefficients for each breast composition.

	Breast composition			
	Fatty	Scattered	Heterogeneous dense	Extremely dense
Number of images	2	26	38	4
Average dice coefficient	0.992	0.832	0.904	0.943

## Discussion

Discussion of results

The average dice coefficient was very high (>0.85) with five or more downsampling layers when semi-subjective corrected region images were used as correct images. For overall accuracy, the average dice coefficient became higher with an increase in the number of downsampling layers from three to six; however, the average dice coefficient became lower when the number of downsampling layers was seven. This can be attributed to the increase in the amount of information that can be obtained with an increase in the number of downsampling layers. However, the accuracy will continue to remain high if the number of layers of downsampling is too large, and therefore, it is necessary to conduct experiments with an appropriate number of layers of downsampling for the number of data to be used. An appropriate combination of parameters results in the highest accuracy of 0.873.

Regarding the orientation of the input images, when the orientation of the input images was simply changed as in Dataset 1 and Dataset 2, the same accuracy was obtained. It is thought that the automatic extraction of mammary gland regions is possible independent of the orientation of the mammograms. When the orientation of the input images was changed at a random rate, as in Dataset 3 and Dataset 4, there was a difference in accuracy. This is because the increase in the number of training patterns presented a challenge to the AI, which may have resulted in a difference in accuracy. Dataset 3 is a dataset in which the mammograms are inverted in the left-right direction at a random rate for the case where the mammograms and the semi-subjective corrected region images are all aligned in the left direction. In this case, the training pattern for one case was moderately increased, which worked as a moderate stimulus for the AI and improved the accuracy compared to Dataset 1 and Dataset 2. Dataset 4 is the case where the mammogram and semi-subjective corrected region images are left-oriented for the left breast and right-oriented for the right breast. Dataset 4 is a dataset in which the mammograms are inverted in the left and right directions at a random rate for the case with the left breast facing left and the right breast facing right. In this case, the training pattern for a single case was more complex and acted as a strong stimulus to the AI, which could not acquire the information well, resulting in lower accuracy compared to Dataset 1 and Dataset 2.

The average dice coefficient for each breast composition was the highest for fatty images. For the scattered to extremely dense images, the higher the mammary gland content, the higher the average dice coefficient. The higher dice coefficient for the fatty images was attributed to the use of unmasked images. Figure 4 shows the unmasked images. The dice coefficient of the black region in the output region-extracted images (Y) was calculated for the black region in the semi-subjective corrected region images (X). The dice coefficients were calculated for the black regions, and therefore, the average dice coefficients were considered to be higher. For the scattered to extremely dense images, the higher the mammary gland content, the higher the average dice coefficient because of the differences in the sizes of the regions to be extracted. Figure 5 shows that the average dice coefficient was about 0.8 for the scattered images with the mammary gland content between 10% and 50%, where each region was scattered like a small island and the regions were complexly structured. For clinical use, cases with scattered mammary glands tend to show lower dice coefficients than other breast compositions. In contrast, cases with extremely dense regions had a mammary gland content of 80% or more, and the regions were simply extracted as a single large island. Therefore, the average dice coefficient was probably as high as about 0.9 because of the simplicity of the region extraction. The high average dice coefficient of 0.9 or higher for “dense breast,” which are more likely to require additional ultrasound examinations in breast cancer screening, would be useful for physicians in deciding whether to add ultrasound examinations. The average dice coefficient is >0.8 for all breast composition, and we believe that this method is clinically useful when considering the characteristics of mammary gland region extraction for each breast composition.

Usefulness of semi-subjective corrected region images

Region extraction was performed using semi-subjective corrected region images. Objective thresholding images were used to create correct region images to confirm the usefulness of the semi-subjective corrected region images. The correct region images used in the 530 images for training and the 70 images for validation were changed to objective thresholding images for U-Net. We used the same parameters as those for the semi-subjective corrected region images (downsampling layers, six; learning rate, 0.0001; and batch size, 16). Seventy images for the test and semi-subjective corrected region images were used as the correct region images. Furthermore, Dataset 3 was used as the orientation of the input images, and the average dice coefficient was calculated. The average dice coefficient was 0.873 when objective thresholding images were used. The average dice coefficient using semi-subjective corrected region images with the same parameters and input image orientation was 0.882, thereby resulting in about 0.01 lower accuracy when the objective thresholding images were used for training.

**Figure 6:**
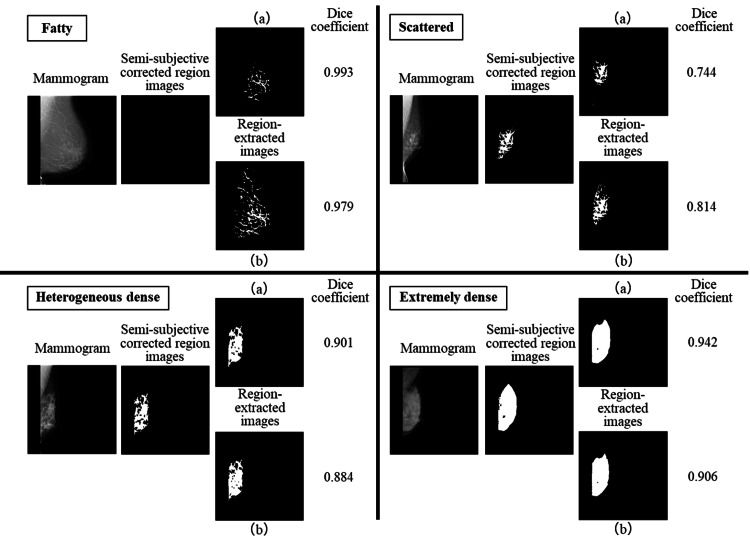
Comparison of semi-subjective corrected region images and objective thresholding images for each breast composition. (a) Semi-subjective corrected image used for training and (b) objective thresholding image used for training.

Table 5 provides a comparison of the average dice coefficients for the semi-subjective corrected region images and objective thresholding images for the overall and individual breast compositions. In the objective thresholding images, regions with blood vessels and pectoral muscles deleted by creating the semi-subjective corrected region image remained, and training was performed with many regions that were extracted as mammary tissues remaining. Thus, the average dice coefficient was higher for the scattered images. Figure 6 shows the differences in the output region extraction images when the semi-subjective corrected region image and objective thresholding image for each breast composition were used for training. The 70 images used for the test were semi-subjective corrected region images. Figure 6a-6b shows the case in which the semi-subjective corrected region image and objective thresholding image were used for training, respectively. The pectoral muscles and blood vessels were output as mammary gland regions when objective thresholding images were used for training. However, only the mammary gland regions were correctly extracted when semi-subjective corrected region images were used for training. Therefore, we believed that semi-subjective corrected region images were useful because they accurately extracted only the mammary gland regions and obtained a high average dice coefficient. The use of semi-subjective corrected region images could reduce the cost and time required to annotate correct regions in segmentation. Moreover, the mammary gland area can be quantitatively determined based on the ability to properly distinguish the pectoralis major muscle, blood vessels, and abnormal findings from mammary gland tissue. In addition, it will be possible to show the quantitative extraction of mammary gland regions to patients when informing them of the breast composition.

**Table 5:** Comparison of semi-subjective corrected region images with objective thresholding images.

	All	Breast composition			
		Fatty	Scattered	Heterogeneous dense	Extremely dense
Number of images	70	2	26	38	4
Semi-subjective correct region images	0.882	0.992	0.832	0.904	0.943
Objective thresholding images	0.873	0.982	0.840	0.889	0.887

Limitation

Approximately 40% of Japanese women of all ages have “dense breasts” [30]. However, the dataset used in this study comprised approximately 40% and 60% of “fatty breast” and “dense breast,” respectively, because we collected data from a single institution over a certain period. In the future, a dataset that is as close as possible to the real number should be created. Furthermore, unmasked images were present in the “fatty” and “scattered” categories because of the difference in timing between the subjective evaluation performed by a breast physician based on the objective threshold processing of images and the evaluation of breast composition. These two processes must be performed simultaneously to eliminate variations in the breast composition of the unmasked images. In future studies, we aim to enhance the accuracy of “scattered,” which exhibited a low dice coefficient among the four classifications of breast composition. Additionally, this study was conducted using data from a single facility and manufacturer, so we need to validate the method using larger datasets.

## Conclusions

We examined the automatic extraction of mammary gland regions using semi-subjective corrected region images. The creation of semi-subjective corrected region images allowed us to define the mammary region more accurately as the correct region compared to subjective judgments. As for accuracy, the average dice coefficients for the overall and per-breast composition resulted in high results. It is clinically significant because the quantitative and accurate extraction enhances confidence in informing patients regarding their breast composition decisions. The use of semi-subjective corrected region images will reduce the cost and time required for the annotation of mammary gland regions.
